# Genomic Landscape of Pleural Mesothelioma and Therapeutic Aftermaths

**DOI:** 10.1007/s11912-023-01479-1

**Published:** 2023-11-28

**Authors:** Alistair Nash, Jenette Creaney

**Affiliations:** 1 National Centre for Asbestos Related Diseases, University of Western Australia, Perth, Australia; 2 Medical School, University of Western Australia, Perth, Australia; 3 Institute for Respiratory Health, Perth, Australia; 4 Department for Respiratory Medicine, Sir Charles Gairdner Hospital, Perth, Australia; 5 The University of Western Australia, Level 5, Harry Perkins Building, QQ Block, QEII Medical Centre, 6 Verdun St., Nedlands, WA 6009 Australia

**Keywords:** Mesothelioma, Genetics, Sequencing, Oncogenesis

## Abstract

**Purpose of Review:**

In this article, we provide a comprehensive analysis of recent progress in the genetic characterisation of pleural mesothelioma, and the translation of these findings to clinical practice.

**Recent Findings:**

Advancements in sequencing technology have allowed the identification of driver mutations and improved our understanding of how these mutations may shape the mesothelioma tumour microenvironment. However, the identification of frequently mutated regions including CDKN2A, BAP1 and NF2 have, to date, not yet yielded targeted therapy options that outperform standard chemo- and immunotherapies. Similarly, the association between mutational profile and the immune microenvironment or immunotherapy response is not well characterised.

**Summary:**

Further research into the link between tumour mutational profile and response to therapy is critical for identifying targetable vulnerabilities and stratifying patients for therapy.

## Introduction

Pleural mesothelioma is a rare malignancy that arises from the mesothelial cells lining the chest. This cancer is primarily associated with asbestos and develops 30–40 years after exposure. Once diagnosed, pleural mesothelioma progresses rapidly; the median survival for patients is approximately 12 months with only 5% surviving to 5 years. However, occasional patients with indolent disease have survived for a decade after diagnosis. Despite the banning of asbestos products in many countries, global rates of mesothelioma diagnosis have increased over time; there are an estimated 30,000–40,000 deaths per year worldwide, but the exact incidence rate is difficult to determine due to differences in reporting between jurisdictions [1]. Australia has a relatively high incidence of 2.5 cases per 100,000 people [2].

There are three major histological subtypes of mesothelioma of prognostic importance: approximately 60% of cases are epithelioid, 20% sarcomatoid and the remainder biphasic which contain combinations of both epithelioid and sarcomatoid histology. Untreated patients with sarcomatoid mesothelioma have a dismal prognosis of 3–4 months. In addition, there are rare architectural patterns, cytologic features and stromal characteristics seen in epithelioid or sarcomatoid mesothelioma that have an impact on patients’ prognosis [3]. Individual tumours can be quite heterogeneous; indeed with increased tissue sampling, many tumours have been reported to comprise a mixture of epithelioid to sarcomatoid histologies.

Histological classification of mesothelioma is useful for therapeutic management. Recently adopted combination immune checkpoint inhibitor (ICI) therapy is favoured for patients with non-epithelioid tumours who obtain a 9-month median increase in survival compared to chemotherapy [4••]. Previously, patients with non-epithelioid mesothelioma benefited little from treatment with either chemotherapy or cytoreductive surgery. Optimal patient selection and approach is under active investigation, with the results of MARS2 eagerly awaited. More studies are needed to determine if immunotherapy is superior to, or synergistic with, chemotherapy in patients with epithelioid tumours [4••, 5].

**Fig. 1:**
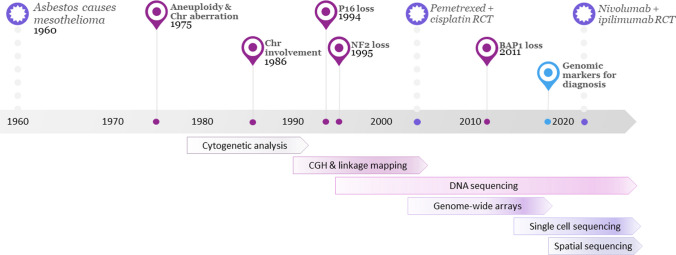
A timeline of the advances in molecular profiling and treatment of pleural mesothelioma

An improved understanding of the oncogenesis and molecular pathogenesis of mesothelioma may lead to an improvement in treatment and patient outcomes. Specific research has focused on understanding the genetic landscape in mesothelioma to identify new diagnostic and prognostic tools, as well as predicting treatment response and the identification of new treatment approaches. In this review, we will describe the key findings from examination of the mesothelioma genetic landscape and discuss how this has and may in the future improve patient outcomes. Increased understanding of mesothelioma biology has been enabled by technological advances, and the introduction of new platforms will hopefully see further increases in knowledge (Fig. 1).

## Mesothelioma is a Cancer of Genetic Loss

Asbestos was recognised in the 1960s as a causative agent for mesothelioma [6]. Subsequent laboratory investigations showed cells exposed to asbestos developed genetic abnormalities including chromosome breakages and aneuploidy [7]. Analyses of the abnormal karyotype of patient derived cell lines suggested some chromosomal regions were commonly altered with deletions, inversions and translocation on chromosomes 1, 2, 3, 6, 9, 11, 17 and 22 [8], as well as monosomy of chromosomes 4 and 22. In 1994, molecular analyses of multiple loci on the short arm of chromosome 9 indicated that alteration of p16/*cyclin dependent kinase inhibitor 2A* (*CDKN2A*) was common in mesothelioma [9]. The following year, common mutations in the *neurofibromatosis type 2* (*NF2*) tumour suppressor gene were identified [10, 11]. Sixteen years later in 2011, focussed sequencing of potential driver genes on chromosome 3p21 demonstrated the *BAP1* gene (encoding the BRCA1-associated protein 1) to be frequently mutated [12]. Clinically, loss of BAP1 and *CDKN2A* expression can be useful ancillary markers for differentiating between cancer and reactive mesothelial cell proliferations [13] and are required for diagnosis of the recently established pathological entity malignant mesothelioma *in situ* (MMIS) [14].

Greater chromosomal instability is associated with poor prognosis [15], and it has recently been shown that whole-genome duplication is significantly associated with decreased overall survival [16]. At a single-gene level, there is strong evidence that homozygous loss of *CDKN2A* is a significant independent adverse prognostic factor [17]; however, there are conflicting data surrounding prognostic significance of somatic BAP1 loss [18]. In a clinical setting, prognosis prediction remains centred around tumour histology and patient performance status.

## BAP1 Tumour Predisposition Syndrome

While asbestos exposure has long been understood as a main cause of mesothelioma, additional significant genetic risk factors have also been identified. In 2011, germline mutations in *BAP1* were observed in two families with a history of cancer, particularly mesothelioma [19]. BAP1 tumour predisposition syndrome (BAP1 TPS) is now recognised as an autosomal dominant condition where individuals carrying heterozygous *BAP1* mutations are at high risk of mesothelioma and other cancers including melanoma [20]. There are notable differences in the clinical behaviour between sporadic mesothelioma and the form of mesothelioma associated with BAP1 TPS, with the latter having a less aggressive phenotype and patients with a higher median overall survival of 5–7 years [21, 22]. The significantly earlier onset of mesothelioma in patients with BAP1 TPS may also be a factor in the better clinical outcomes seen for these patients.

With increasing scrutiny, the list of potential pathogenic germline variants associated with mesothelioma is expanding. So far, potential mutations have been identified in over 80 genes, including in known tumour suppressor genes and genes associated with other hereditary cancer syndromes, DNA maintenance and repair as well as genes commonly somatically mutated in mesothelioma (review in [23]) [24]. The prevalence of pathogenic germline variants in the entire mesothelioma cohort is difficult to estimate, given that many of the studies designed to identify such genetic susceptibility markers have been in cohorts enriched based on having a family history of cancer. The identification of mutations associated with mesothelioma susceptibility raises the possibility of screening individuals at high risk of developing mesothelioma (and other cancers) with the goal of identifying early-stage disease. However, there are a myriad of clinical, ethical and economic implications that need to be considered before this becomes a widely adopted practise.

## Mesothelioma Prognosis

At the turn of the century, the increased general availability of array-based technologies, especially for the evaluation of gene expression, saw a large number of studies comparing gene expression between (i) non-malignant mesothelial cells and malignant mesothelioma, (ii) different mesothelioma histologies, and (iii) different prognostic outcome groups. These studies generated a number of candidate genes, gene-expression signatures and gene ratios that were proposed for use in various clinical settings, particularly as prognostic predictors. Interestingly, there was a general lack of overlap in the gene lists from different studies. In some, but not all studies, the gene expression–based prognostic signatures performed better than predictors based on clinical characteristics and tumour histology.

More recently, such comparative analyses have been expanded upon, incorporating data from multiple ‘omics platforms to perform a comprehensive analysis from combined gene expression, mutation profile, copy number, DNA methylation, microRNA expression and other datasets (Table 1). The benchmark papers reflect this approach arising from the Boston group [25] and from the international efforts coordinated by The Cancer Genome Atlas (TCGA) [26]. Based on gene expression data, Bueno and colleagues classified mesothelioma into four clusters which reflected the continuum of established tumour histology i.e. sarcomatoid, biphasic-sarcomatoid (biphasic-S), biphasic-epithelioid (biphasic-E) and epithelioid clusters. The epithelioid cluster had better overall survival than the other clusters. None of the epithelioid cluster of cases had mutated *TP53*; the gene encoding the well-known tumour suppressor p53 and *TP53* mutation itself were associated with poor prognosis [25]. Comparison of clusters derived from different mesothelioma cohorts by differing methodologies from different ‘omics platforms shows that the two main groups of highly correlated clusters present in all datasets are associated with epithelioid and sarcomatoid histology [27].

**Table 1:** Summary of recent studies utilising integrated ‘omics to examine the pleural mesothelioma microenvironment

Year	Lead Author	Descriptor	Number of cases^a^	SNP array	DNA sequencing	RNA	Methylation	RPPA	Phenotypic
2023	Nair [28]	NCI	122	No	WES	100	No	No	Yes
2023	Mangiante [58]	MESOMICS	120	No	WGS	109	119	No	Yes
2022	Hiltbrunner [38]		1468 (1113 pleural)	No	Targeted F1CDx and F1	No	No	No	No
2022	Dagogo-Jack [30•]	FMI	1294 (980 pleural)	No	Targeted F1CDx and F1	No	No	No	No
2022	Creaney [16]		58	No	WGS	58	No	No	Yes
2021	Nastase [59]		118	121	Combination (WGS; WES; Targeted)	35	No	No	Yes
2021	Zauderer [29]	MSK-IMPACT	194	No	Targeted MSK-IMPACT	No	No	No	Yes
2019	Blum [60]		63	No	No	63	62	30	Yes
2018	Hmeljak [26]	TCGA	74	No	WES	74	74	52	Yes
2016	Bueno [25]		216	95	Combination	211	No	No	Yes

*RPPA* reverse-phase protein array

^a^Number stated in abstract, does not necessarily reflect number of cases used in each subset analysis

At the time of writing, the most recent prognosis signature reported consisted of 48 genes that when overexpressed were associated with poor prognosis [28]. This prognostic signature was validated using both the Bueno and TCGA cohorts. Among the 48 gene signatures, the expression of one gene in particular, *CCNB1*, which encodes cyclin B1, was, after controlling for age and sex, associated with worse prognosis in all three cohorts [28].

Machine-learning approaches are increasingly being applied to mesothelioma datasets to identify molecular subtypes and define prognostic algorithms. For example, a patient-level prognosis tool known as OncoCast-MPM was generated using data from mutational profile, clinical features and pathology data, and validated in the TCGA cohort [29]. Currently though, prognostication tools based on genomic data have not been incorporated into routine patient care; thus, tumour histology and stage remain the major considerations for risk stratification in mesothelioma patients.

## Frequently Altered Genes in Mesothelioma Do Not Reveal Actionable Drivers

One of the main drivers for comprehensive genomic characterisation is to uncover the biological mechanisms and drivers of oncogenesis. In non-small cell lung cancer (NSCLC), this has resulted in dramatic clinical advances and a more personalised approach to patient care. While NSCLC is characterised by activating mutations in oncogenes, mesothelioma on the other hand is characterised by the frequent inactivating alteration of the *CDKN2A*, *NF2* and *BAP1* tumour-suppressor genes. A recent cataloguing of mutations in 980 pleural mesothelioma samples revealed that 10% or greater of cases had alterations in *CDKN2A* (49%); *BAP1* (44%); *CDKN2B* (42%); *MTAP* (34%); *NF2* (33%); *TP53* (18%) and *SETD2* (10%). The frequent alteration in *CDKN2A*, *CDKN2B* and *MTAP* reflects their co-localisation of chromosome 9p21 [30•]. Genetic alterations have also been frequently observed in the *TERT* promoter [27].

Rare cases of mesothelioma have been described with mutations in *KRAS*, *NRAS* [27] and potential oncogenic fusions involving *ALK* and *EWSR1* rearrangements [31–33], as well as inactivating mutations in *VHL* raising the possibility of targeted therapy in such cases [34]. The recent cataloguing paper reported no ALK rearrangements and that 2% of pleural mesotheliomas had activating mutations in *KRAS*. However, the authors raised the possibility, given that their study had limited correlatory data that some cases may have been mis-diagnosed lung cancers and not mesothelioma [30•].

The scarcity of actionable mutations in mesothelioma limits the application of targeted drug treatments, and therefore, many trials have focussed on pathways affected by genetic alterations affecting *BAP1*, *CDKN2A* and *NF2* (reviewed in [35]). Overall, results from these trials have been modest with response outcomes in line with historical controls. However, on-going and future trials look to exploit the effects of combinatory treatment approaches. Our own recent work suggests that genetic alteration of *BAP1* may be a predictive biomarker for overall prognosis and survival after combination pemetrexed and platinum chemotherapy. In two independent clinical cohorts from Australia and Denmark, we observed significantly longer median survival in those with loss of BAP1 protein expression after first-line treatment, i.e. Australian cohort 19.6 versus 11.1 months (*p* < 0.01) and Danish cohort 20.1 versus 7.3 months (*p* < 0.001) [36].

## Tumour Mutational Burden and Immune Profiling

In this era of immunotherapy, there is intense interest in predicting which patients are likely to benefit and which experience adverse side effects following immune-based treatments. A high tumour mutational burden (TMB) is recognised as a predictor of good outcomes with immunotherapy, and this has been translated into clinical practise with the FoundationOne CDx assay defined cut-off of > 10 mutations per mega base (mutations/Mb), approved by the US Food and Drug Administration as a companion biomarker for selecting patients for pembrolizumab immunotherapy [37]. Using the FoundationOne assay, Dagogo-Jack showed in 980 pleural mesothelioma cases a median of 1.74 mut/Mb, and that microsatellite instability which often results in high TMB was only present in one case in the series [30•]. An expanded cohort which included an additional 123 pleural cases confirmed these findings [38]. Therefore, in contemporary practice, there is little value in this standard definition of TMB for stratifying mesothelioma patients for immunotherapy, and other approaches to identify biomarkers for immunotherapy response are actively being investigated.

To date, only limited data linking immunotherapy treatment outcomes to genomic/transcriptomic data is available in mesothelioma. Therefore, gene expression data from non-immunotherapy treated cohorts has been used to estimate tumour-infiltrating lymphocytes and to characterise the tumour microenvironment to determine if they can serve as biomarkers for overall survival. Results from within and across cohorts have highlighted the heterogeneity and complexity of the mesothelioma tumour microenvironment [16, 39, 40, 41•, 42]. Several analyses of cohort and publicly available data, some using machine learning–based integrated methodologies, have identified sub-types of mesothelioma based on immunological profiles. The study by Alay and colleagues proposed three sub-groups, with the group characterised by high cytotoxic T cell and low T-helper cell gene signatures having the best prognosis [39]. Another study showed that an inflammatory environment with high B cell numbers as well as tertiary lymphoid structures was associated with better prognosis [40]. In a further study, cases could be divided into immune-related or non-immune groups, with the immune group being further classified as either immune-suppressed or immune-activated. The immune-suppressed mesothelioma group accounted for approximately 20% of the cohort and was enriched with stroma, myeloid components and immunosuppressive macrophages; the group had poor clinical outcomes, in both the discovery data set and the TCGA dataset used for validation [42]. Another recent analysis using the popular deconvolution algorithm CIBERSORT, which estimates the abundance of 22 immune cell subsets from gene expression data, found that M2 macrophages were associated with unfavourable prognosis in the NCI dataset, but not in the TCGA or Bueno datasets [28]. Overall, these studies reinforce the message seen in cancer in general that immunologically ‘hot’ or ‘active’ tumours, however they are defined in a given study, have better prognosis. However, in mesothelioma, this group of immunologically active tumours tends to be the smallest of any cohort. We note that care should be taken with the use of the TCGA mesothelioma dataset to ensure that only the final set of 74 cases is used in analysis [26], as the inclusion of 13 cases in the pre-release data set that were excluded from the final analysis in such a small cohort could significantly confound results.

Recent analyses of mesothelioma patients treated with immunotherapy are looking to identify biomarkers that can predict treatment efficacy. In lung cancer, PD-L1 expression is a predictive biomarker of PD-1 or PD-L1 immunotherapy response; however, this association was not found in mesothelioma in either the seminal CHECKPOINT-743 nivolumab plus ipilimumab phase 3 study [4••]; the CONFIRM trial of nivolumab [43] or the two Phase 2 durvolumab plus chemotherapy studies (DREAM [44] and PrE0505 [45•]). Exploratory analysis in PrE0505 suggested that high immunogenic mutation burden and diverse T cell repertoire may be linked to favourable outcomes [45•]. In the CHECKMATE-743 cohort, a known four-gene inflammatory signature score, which measured expression of *CD8A*, *STAT1*, *LAG3* and *CD274*, was correlated with improved survival in the immunotherapy-treated arm but not the chemotherapy arm [46]. Despite this latter encouraging finding, the authors emphasised that additional work is needed to identify and validate treatment predictive biomarkers. Interestingly, there are some data suggesting that some of the common genetic alterations may be associated with particular immune environments, for example, in a small study, loss of BAP1 in peritoneal mesothelioma correlated with an inflammatory tumour environment [47], and CDKN2A deletion was associated with a PD-1-resistant phenotype [48].

## Single-Cell Sequencing, Spatial Transcriptomics and Artificial Intelligence

Advances in understanding of mesothelioma that have led to improvements in outcomes for mesothelioma patients have in part paralleled advances in technologies that enabled study of the underlying genomic landscape. The next wave of accessible technologies includes those to study not only the transcriptome but also the genome, epigenome and proteome at a single cell level, and study genomic events in the context of tissue architecture.

To date, in mesothelioma, single-cell transcriptomic (scRNA-seq) studies are limited. In a study of five pleural effusions and tumour samples, a subpopulation of macrophages was identified which may be targetable with specific therapies [49]. Using a mouse mesothelioma model, an inflammatory monocyte subpopulation was identified that was associated with immune checkpoint therapy response [50]. However, scRNA-seq data is being rapidly generated across cancer types [51], with work examining the single-cell components of the lung producing a reference atlas for heath and disease [52], including non-small cell lung cancer [53]. Individual studies generally integrating single-cell and bulk RNA-seq have revealed subpopulations of cells associated with clinical features such as prognosis [54] or treatment response [55]. Across cancer-types, single-cell transcriptomic studies have enabled more detailed classification of cell types revealing heterogeneity between cells that potentially contributes to cancer development and response to therapy, as well as increasing understanding of intercellular crosstalk, but challenges remain with the technology and our ability to interpret the data generated.

Similarly, there have not been many studies published exploring the spatial transcriptomic platform utilisation in mesothelioma. To date, only a pilot study in mesothelioma has been performed [56] showing the potential of the technology whist demonstrating some of its current limitations relating to individual cell identification, and the limited software ecosystem that currently lacks well-established bioinformatic workflows for analysing the data. In addition, the perennial issues associated with only analysing a small proportion of an often heterogeneous tumour is still a major limitation.

The adoption of artificial intelligence and machine learning-based analyses to available data is likely to improve our understanding and has already become apparent utilising such approaches to the analysis of standard diagnostic pathology slides. Using deep convolutional neural networks, a French-American start-up company Owkin has produced a method, MesoNet, to accurately predict the overall survival of mesothelioma patients from whole-slide digitized images, without any pathologist-provided locally annotated regions. They found that stromal regions associated with inflammation, cellular diversity and vacuolization contributed significantly to patient outcomes [57]. Deep learning and associated models have the potential to identify new features predictive of patient outcomes and to new biomarker discoveries.

## Conclusion

Studies of the genomic landscape of mesothelioma have already identified markers for diagnosis and prognosis, and it is likely in the near future to help with treatment or personalised therapeutic approaches. With the adoption of immunotherapies, it is even more crucial to identify and validate biomarkers of treatment response. Through accurate and personalised analyses, it may be possible to provide individualised patient care, prioritise high-risk patients for clinical trials, evaluate real-world data and generate accurately matched historical control groups.
